# Molecular characterization and high prevalence of *Tritrichomonas foetus* in bulls from the North-West Province of South Africa using real - time polymerase chain reaction (PCR) and conventional PCR diagnostics

**DOI:** 10.14202/vetworld.2025.4129-4145

**Published:** 2025-12-27

**Authors:** Afaque H. Syed, Mpinda Edoaurd Tshipamba, Ngoma Lubanza, Baitsholetsi G. Mokolopi, Jean Marie Dibungi Luseba, Mulunda Mwanza

**Affiliations:** 1 Department of Animal Health, School of Agriculture, Faculty of Natural and Agricultural Sciences, Mafikeng Campus, North-West University, Private Bag X2046, Mmabatho, 2735, South Africa; 2 College of Agriculture and Environmental Sciences, Department of Agriculture and Animal Health, University of South Africa, Florida Science Campus, Johannesburg, South Africa; 3 University of Kinshasa, Faculty of Veterinary Medicine, Democratic Republic of Congo

**Keywords:** *Tritrichomonas foetus*, RT-PCR, conventional PCR, phylogeny, bovine trichomonosis, South Africa

## Abstract

**Background and Aim:**

Bovine trichomonosis, caused by *Tritrichomonas foetus*, is a significant reproductive disease that impacts cattle productivity and breeding efficiency. In South Africa, routine diagnostic methods often depend on culture and microscopy, which may not accurately distinguish *T. foetus* from nonpathogenic trichomonads. This study aimed to determine the prevalence of *T. foetus* in bulls from the Dr. Segomotsi Ruth Mompati (DSRM) District, North-West Province, South Africa, using advanced molecular diagnostics, including real-time polymerase chain reaction (RT-PCR), conventional PCR, DNA sequencing, and phylogenetic analysis.

**Materials and Methods:**

A total of 239 sheath wash samples were collected between June 2018 and October 2020. Of these, 51 culture-positive trichomonad isolates were selected for molecular analysis. Microscopy and modified Giemsa staining were used to characterize protozoal morphology. DNA was extracted and subjected to RT-PCR with 5’ TaqMan™ probes, as well as conventional PCR targeting the 5.8S rRNA/Internal Transcribed Spacer (ITS) regions. PCR amplicons were sequenced, and phylogenetic trees were constructed using MEGA (maximum-likelihood, 1,000 bootstrap replicates). Statistical comparisons between diagnostic methods were performed using Chi-square and Cochran’s Q test.

**Results:**

RT-PCR detected *T. foetus* in 80.4% (41/51) of the culture-positive samples, with most isolates showing low Ct values, indicating strong positivity. Conventional PCR successfully amplified 12 isolates (300–340 bp), all of which were confirmed as *T. foetus* by sequencing. Phylogenetic analysis showed that the isolates clustered with the Southern African genotype, exhibiting 77%–87% similarity to Namibian strains and were closely related to Australian and Turkish isolates. No significant correlation was found between geographic location and PCR positivity. RT-PCR demonstrated significantly higher sensitivity than conventional PCR (p < 0.05).

**Conclusion:**

This study confirms a high prevalence of *T. foetus* in bulls in the DSRM district and demonstrates the superior accuracy of molecular diagnostics compared with culture and microscopy. The identification of genotypes closely related to Southern African strains highlights potential transboundary spread. Incorporating PCR-based screening into routine surveillance is essential for accurate diagnosis, minimizing unnecessary culling, and enhancing reproductive herd health. Further longitudinal studies are recommended to assess disease dynamics and inform regional control programs.

## INTRODUCTION

**Figure 1:**
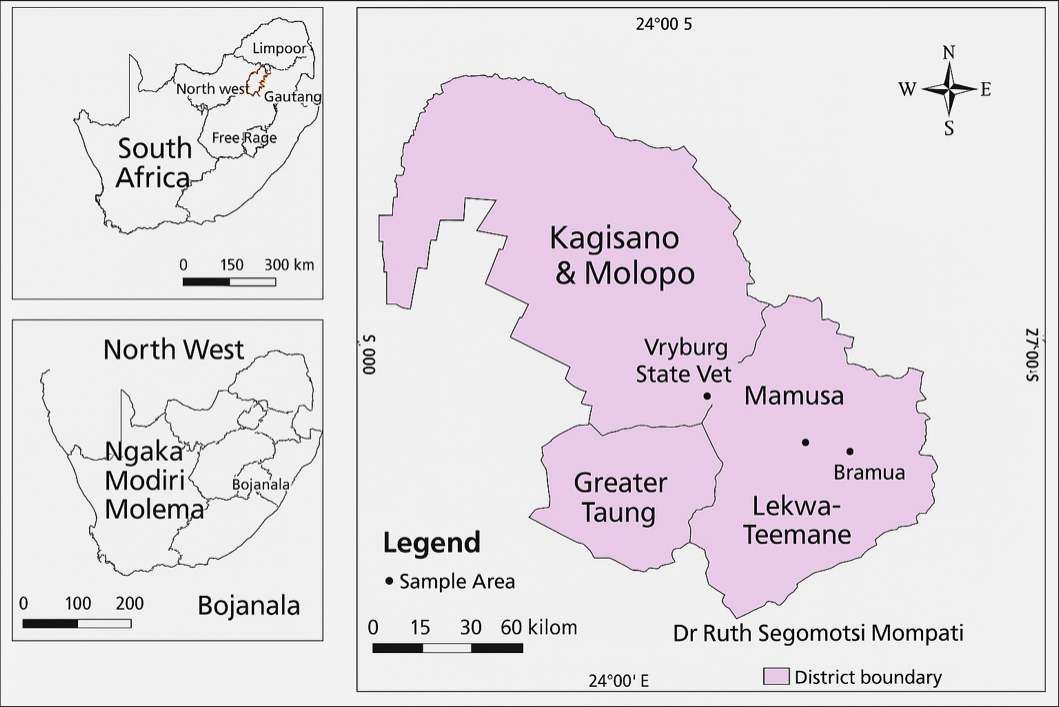
Map showing the different municipalities in the Doctor Segomoti Ruth Mompati region [The map was generated using **QGIS 3.44**].

An extensive range of animals, including humans, serve as hosts to various species within the order *Trichomonadida* [1]. These anaerobic protozoa belong to the family *Trichomonadidae* and the order *Trichomonadida*, specifically the phylum *Parabasalia*, which includes species of both medical and veterinary importance [2]. Members of this phylum have a commensal or parasitic relationship with their hosts in the lower gastrointestinal system [2]. A ranking system classified these trichomonads as Excavata: Parabasalia: Trichomonadida [3]. It is important to note that the trichomonad most commonly encountered in veterinary medicine is the protist of the bovine reproductive tract known as *Tritrichomonas foetus*, which causes the reproductive disease called bovine trichomonosis [4, 5]. Cattle farming is a vital part of the South African economy, especially in regions where natural breeding is prevalent, which inadvertently facilitates the transmission of reproductive diseases [6]. Infertility and abortions caused by sexually transmitted diseases in cattle result in significant economic losses globally, affecting productivity and genetic improvement programs [7, 8]. Widespread commercial and communal beef production systems are sustainable options for farmers in South Africa [9] and are important for food security [10–12]. Although the agricultural sector’s contribution to South Africa’s gross domestic product declined from 3.9% in 1994 to 2.2% in 2017 [13], the Bureau for Food and Agricultural Policy [14] estimated a 25% increase in average annual beef consumption among South Africans in 2020. Bovine trichomonosis tends to reduce cattle productivity by causing losses due to decreased conception rates of less than 50%, leading to longer calving intervals [15]. This infection also causes economic losses due to the costs associated with replacing bulls. Ultimately, sexually transmitted diseases (STDs) primarily cause significant genetic potential loss by decreasing calf viability. The STD caused by the flagellate protist *T. foetus*, shown in Figure 1, is known as bovine trichomonosis [16, 17]. Therefore, the term bovine trichomonosis will be used to refer to the disease caused by *T. foetus* in this manuscript. Bovine trichomonosis is a major reproductive disease in South Africa [8].

In many countries, such as the USA, the current diagnostic protocols mainly focus on analyzing sheath wash samples using either microbial culture or polymerase chain reaction (PCR) analysis [18]. The PCR enables the detection of DNA associated with *T. foetus*, making the avoidance of cross-contamination in diagnostic samples crucial [19]. Previously, the diagnostic method involved collecting sheath wash or vaginal secretions, followed by a single microbial culture in an appropriate *Tritrichomonas* growth medium and microscopic inspection. However, organisms other than *T. foetus* found in samples may lead to inaccurate results. It remains challenging for observers lacking adequate experience in protozoal morphology to accurately distinguish between different trichomonad species. Additionally, factors such as low parasite numbers, prolonged transport times, unfavorable sampling conditions, and varied processing methods can influence clinical sample results. According to Mendoza-Ibarra *et al*. [20], contamination with intestinal or coprophilic trichomonads can impact the diagnosis of *T. foetus* based on selective cultivation. Collectively, these diagnostic challenges, along with current protocols in South Africa, may lead to healthy breeding bulls being mistakenly sent for slaughter as *T. foetus*-positive, even when they carry non-*T. foetus* trichomonads. Since its discovery in South Africa, bovine trichomonosis has affected large cattle herds [21]. However, no isolates from South Africa have yet been compared molecularly, despite their widespread presence across the African continent [22]. According to Collantes-Fernández *et al*. [23], significant economic losses result from low pregnancy rates, extended calving seasons, and reduced calf crops in cows and heifers infected with *T. foetus*. Eradicating *T. foetus* from infected herds remains costly due to the absence of approved treatments, control measures, and the common practice of culling infected animals [24, 25]. *T. foetus* is endemic in regions of South Africa where beef herds depend on natural breeding, particularly in livestock systems owned by economically disadvantaged communities with limited access to veterinary services [26]. Countries that have successfully eradicated the parasite have implemented strict regulations to prevent its reintroduction [27]. Bovine trichomonosis is a notifiable disease listed by the World Organization for Animal Health [28]. In South Africa, some public and private laboratories rely solely on culture methods (e.g., Vryburg State Veterinary Laboratory), while others (e.g., OVI) have incorporated PCR for *T. foetus* detection. A study by Zangure [29] indicated that laboratories using only culture had sensitivities ranging from 63% to 100%, whereas those using PCR reported sensitivities from 81% to 100%. In this study, samples were obtained from the Vryburg State Veterinary Laboratory. After analysis and confirmation of positive results through microbial culture and direct microscopy, no additional confirmatory tests, such as PCR, were performed on the isolates. Furthermore, a study by Casteriano *et al*. [22] identified a new *T. foetus* genotype in Southern Africa, highlighting the importance of fully understanding the diversity and origins of the trichomonad causing bovine trichomonosis in the region. This is the first study to molecularly characterize trichomonads in bulls from the Dr. Segomotsi Ruth Mompati (DSRM) District, North-West Province, South Africa, by investigating the presence of trichomonad species responsible for reproductive losses in bovines within the study area.

The susceptibility to bovine trichomonosis is reportedly influenced by factors such as breed, sex, and age. According to Rae *et al*. [30], there is a higher occurrence of *T. foetus* among Bos taurus (Simmental, Charolais, and Angus breeds) than among *Bos indicus* cattle (Taurus breed). Disease transmission occurs predominantly through coitus between an infected bull and a susceptible cow, as well as through contaminated equipment during artificial insemination (AI) and poor sample collection techniques [22, 31]. A previous study by Ondrak [32] reported *T. foetus* infections on a large Californian dairy farm due to improper hygiene during gynecological examinations and AI using contaminated gloves and instruments. The use of contaminated semen can also spread *T. foetus*. According to Ondrak [32], processed frozen semen lacking proper hygienic procedures, as outlined by the Certified Semen Services (CSS) protocol, may cause *T. foetus* infection [26]. Moreover, Jeffries and Harris [33] state that freezing the protozoa for up to 6 months does not affect their virulence or their ability to cause lesions in mice injected subcutaneously with *T. foetus* [27]. Suspected transmission by flies has been reported; however, there is insufficient evidence to confirm this [34]. It has also been shown that the distribution of *T. foetus* in South Africa varies across regions, with prevalence rates ranging from 0.9% in Western Transvaal to 26.4% in the former Republic of Transkei. In Thabazimbi (Limpopo Province), the prevalence is recorded at 6.2% using direct microscopy, while the former Orange Free State (southern part) shows a lower prevalence of 3.7% with the same diagnostic method [30, 35]. Western Transvaal exhibits the lowest prevalence at 0.9%, which can also be detected via direct microscopy [35]. In contrast, molecular diagnostic methods such as PCR have been employed in some areas, including an unspecified region in South Africa (4.5%) and the Magaliesburg area in Gauteng (2.1%), using sheath washings and scrapings [36, 37]. The significant variation in prevalence across these regions may be attributed to differences in diagnostic techniques, sampling methods, and regional epidemiological factors [38]. Transmission of *T. foetus* occurs mainly through coitus, involving infected bulls and vice versa. Additionally, contaminated equipment during AI and poor sample collection techniques contribute to the spread [22, 31]. A prior study by Ondrak [32] reported *T. foetus* infections on a large Californian dairy farm due to improper hygiene during gynecological exams and AI using contaminated gloves and instruments. Contaminated semen can also transmit *T. foetus*. As noted by Ondrak [32], processed frozen semen that does not adhere to proper hygienic procedures, such as those outlined by the CSS protocol, may cause infection [26]. Furthermore, Jeffries and Harris [33] state that freezing the protozoa for up to 6 months does not alter their virulence or their capacity to cause lesions in mice injected subcutaneously with *T. foetus* [27]. Suspected transmission by flies has been reported; however, there is insufficient evidence to confirm it [34].

Despite the long-standing recognition of bovine trichomonosis in South Africa, a significant gap remains in accurately molecularly characterizing and understanding the epidemiology of *T. foetus* within local cattle populations. Current diagnostic practices in many parts of the country mainly rely on culture and microscopic examination, methods that are susceptible to false positives due to the presence of morphologically similar non-*T. foetus* trichomonads. Only a few South African laboratories use PCR-based methods, and even fewer perform confirmatory sequencing, leading to considerable uncertainty about the actual prevalence, strain distribution, and genetic diversity of *T. foetus*. Notably, no comprehensive molecular comparisons of isolates from the North-West Province have been conducted, despite the region’s heavy reliance on natural breeding and its proximity to Namibia and Botswana, areas linked to identified Southern African genotypes. This lack of molecular data hampers the ability to differentiate pathogenic *T. foetus* from nonpathogenic trichomonads, impedes evidence-based disease surveillance, and limits the development of region-specific control strategies. Additionally, there is a substantial knowledge gap regarding how different diagnostic techniques compare in terms of sensitivity and specificity under field conditions in South Africa. Collectively, these issues highlight the urgent need for molecular epidemiological studies that validate diagnostic methods, characterize circulating genotypes, and provide accurate prevalence estimates to support informed reproductive health management in cattle.

The current study aimed to fill these knowledge gaps by conducting a comprehensive molecular analysis of trichomonad isolates collected from bulls in the DSRM of South Africa’s North-West Province. Specifically, the study sought to validate diagnoses based on culture and microscopy using advanced molecular techniques, such as RT-PCR, conventional PCR, and DNA sequencing. Additionally, the research aimed to determine the prevalence of *T. foetus* in the area, compare diagnostic results from different laboratory methods, and perform phylogenetic analysis of the isolates to assess their genetic relationship to previously reported Southern African and international genotypes. By combining traditional and molecular diagnostic methods, the study aimed to produce accurate, region-specific epidemiological data, clarify the identity of trichomonads in bovine reproductive samples, and provide essential information to improve diagnostic accuracy, disease monitoring, and reproductive management strategies in South African cattle

## MATERIALS AND METHODS

### Ethical approval

Ethical approval was obtained from the Animal Care, Health, and Safety in Research (AnimCare) committee at North-West University. The ethics approval number for this study was NWU-01882-19-A5. Additionally, permission to conduct this study was secured from the Department of Agriculture, Forestry, and Fisheries in accordance with Section 20 of the Animal Disease Act (ACT: 35 of 1984).

### Study period and location

The study was conducted from June 2018 to October 2020 in the DSRM municipality of the North-West Province (Figure 1), which was formerly known as the Bophirima District Municipality. DSRM is one of the four districts in the North-West Province of South Africa. Located along the borders with Botswana and Namibia to the north and the Kalahari Basin to the South, the area spans over 2 million km².

### Study design

This study is the first to provide a molecular characterization of Tritrichomonas isolates from the Dr. Segomotsi Ruth Mompati District, using a cross-sectional approach to determine *T. foetus* prevalence with advanced PCR techniques. The laboratory is SANAS-accredited for culturing and identifying *T. foetus* (facility number V0038, accreditation number ISO/IEC 17025: 2005).

### Sample size determination

As the total number of bulls in the study area was unknown at the time of sample collection, a statistical formula for an infinite population was applied to determine the sample size:

- Z = projected standard deviation at a 95% confidence interval (Z = 1.96)
- SD (Standard deviation) = 15%
- = estimated error at 5%

Thus:

n = ([1.96]² × [0.15]²) / ([0.05]²)n = 34.6 × 5 (local municipalities) = 173 samples

A total of 173 clinical samples were needed for this analysis. The Vryburg Veterinary Laboratory received 239 sheath wash samples, distributed as follows: Naledi (144), Mamusa (18), Kagisano-Molopo (65), Greater Taung (4), and Lekwateemane (8). Out of these, 51 samples suspected of being positive for *T. foetus* were selected for DNA extraction and PCR. The 51 isolates were distributed as follows: Naledi (21), Mamusa (4), Kagisano-Molopo (19), Greater Taung (4), and Lekwateemane (3).

### Sample collection and transportation

This study presents a rigorous two-step diagnostic method that combines traditional microscopic evaluation with advanced molecular diagnostics to enhance detection specificity and decrease false positives in *T. foetus* identification. After initial screening at the Vryburg Provincial Veterinary Laboratory, 51 *T. foetus*-positive cultures confirmed microscopically were given unique identifiers (NWT 1–51) for molecular analysis. The samples were kept at 4°C during transport using Steve’s transport media obtained from Onderstepoort Biological Products (OBP), RSA, and were sent to the Animal Health Laboratory, Faculty of Natural and Agricultural Sciences, North-West University, Mafikeng Campus.

### Isolation and identification of Tritrichomonas

#### Protozoal culture

Unlike previous studies that relied only on standard culture methods, this study used a controlled re-culturing protocol with optimized Diamond’s medium (Batch number: 3111815; Mediamage™ South Africa), ensuring better protozoal viability and improved diagnostic accuracy. The inoculated cultures were incubated at 37°C for 7 days or until visible growth appeared.

#### Wet-Slide preparation technique

Twenty-five μL of fresh culture was placed on a labeled glass slide (Sail brand, China, Cat. No. 7101) and covered with a coverslip (Cat. No. 470816). Slides were examined under an Olympus BX53 microscope with the condenser iris partially closed and the light intensity adjusted for contrast. Scanning was conducted in a grid pattern for 5–10 min using the ×40 objective. Identification criteria included motile, jerky, rolling movements, pear-shaped morphology, an axostyle extending beyond the posterior end, a prominent undulating membrane, and motile flagella [22].

#### Staining of protozoa

**Figure 2:**
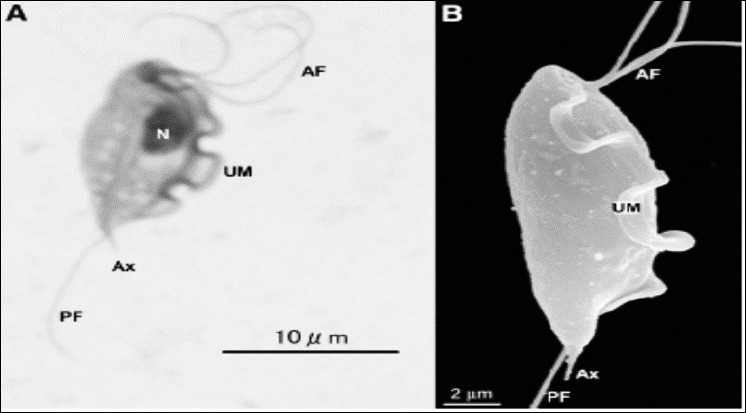
Microscopic images of *Tritrichomonas foetus* trophozoites. The images depicted here are (A) Giemsa stain and (B) scanning electron microscopy images of *T. foetus*. UM = Undulating membrane, AF = Anterior flagella, P = Posterior flagellum.

For improved visualization, staining followed the method of Lun and Gajadhar [39], omitting Lugol’s iodine. Ten μL of culture was smeared onto microscope slides, air-dried, and fixed with methyl alcohol (Diff-Quick kit) for 1 min. Slides were then stained sequentially with eosin (1 min), xanthene dye solution (1 min), and thiazine dye solution (30 s), with rinsing between steps. After drying, slides were examined and photographed at ×100 magnification, as shown in Figure 2.

### Molecular analysis

#### Genomic DNA extraction

An optimized genomic DNA extraction protocol tailored for protozoal samples was used to improve DNA yield and quality. DNA concentrations were measured with a Bio-Rad Nanodrop (USA), and samples were stored at −80°C for further analysis.

#### Genomic DNA amplification

DNA from suspect-positive isolates was tested with both RT-PCR and conventional PCR [40] using the *Tritrichomonas-*specific primer set TFR-1 and TFR-2 (Table 1).

**Table 1:** Primer used for 5.8S rDNA amplification.

Primer	Sequence 5’–3’	Reference	Amplifcation conditions
Forward TFR1	5′- GTA- GGT- GAA- CCT- GCC-GTT-G- 3’	[40]	Thirty s at 94°C for denaturation. Twenty seconds at 58°C for annealing.
Reverse TFR2	5′- ATG- CAA- CGT- TCT- TCA- TCG- TG- 3’	[40]	Thirty s at 72°C for extension. 1200 s at 72°C for final extension.

#### RT-PCR screening

All 51 suspect-positive isolates were tested using 5’ TaqMan™ DNA probes (Applied Biosystem, USA) as described by McMillen and Lew [41]. RT-PCR reactions (20 μL) included DNA template, FAM-labeled probes, 2× Precision Plus Master Mix, an internal amplification control (IAC), and nuclease-free water. Reactions were run on a StepOne Plus™ RT-PCR system.

This is the first study in the region to use a 5′ TaqMan™ RT-PCR assay with minor groove binders (MGB) for *T. foetus*, offering superior specificity. Thermocycling conditions included 95°C for 2 min, followed by 50 cycles of 95°C for 10 s and 60°C for 60 s. Fluorescence was detected through 6-carboxyfluorescein (FAM) and Applied Biosystems VIC fluorescent dye (VIC)(6-carboxyfluorescein) and VIC channels. IAC DNA ensured reaction validity and reduced inhibition [40].

#### Conventional PCR and gel electrophoresis

Conventional PCR was conducted on all 51 isolates using an Engine DYAD Peltier thermal cycler (Bio-Rad, USA). Reaction volumes (50 μL) contained Master Mix (#10050007 Biolabs-SA), DNA template, nuclease-free water, and primers. Amplicons were separated on a 1.5% agarose gel, stained with ethidium bromide, and visualized with a Syngene Ingenious Bio Imager. Confirmed *T. foetus* MT750332 served as the positive control.

### DNA sequence analysis

PCR products were sequenced using an ABI PRISM® 3500XL DNA sequencer (Inqaba Biotechnology). This study provides new sequence data from South African *T. foetus* isolates, enhancing the global understanding of strain diversity. BLAST (Basic Local Alignment Search Tool) was used to identify similar sequences [42], which were aligned with ClustalW [43] and edited in BioEdit 7.2 (https://bioedit.software.informer.com/7.2/). Maximum parsimony analysis was conducted using DNA Pair [40], with results bootstrapped at 1000 replications.

### Phylogenetic data analysis

Multiple sequence alignment was carried out using MAFFT 6.864. Evolutionary distance matrices were generated [44]. Phylogenetic trees were built using Neighbor-Joining and maximum-likelihood methods in MEGA 5.10 [44, 45]. Bootstrapping with 1000 replicates assessed the reliability of the trees [46]. Putative chimeric sequences were identified with ChimeraBuster 1.0, and tree visualization and editing were performed using TreeView [47].

### Statistical analysis

Frequency tables and pie charts summarized the sample distribution and diagnostic outcomes (conventional PCR vs. RT-PCR). Cross-tabulations and clustered bar charts compared incidence by municipality and diagnostic method. Chi-square tests evaluated the association between municipal location and *T. foetus* occurrence, with significance set at p < 0.05. Additionally, Cochran’s Q test was used to assess differences in detection rates between conventional PCR and RT-PCR, ensuring methodological robustness. A significant p-value indicated that the results from the two methods differed notably.

## RESULTS

### Morphological analysis based on culture and microscopic examination

**Figure 3:**
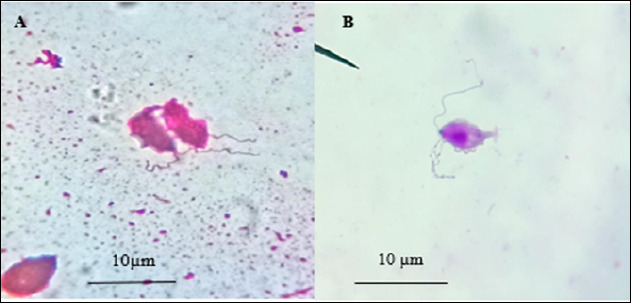
Giemsa-stained slides (x100) of isolated trichomonads from the bovine preputial cavity: trichomonads (A) with three anterior flagella and (B) with four anterior flagella.

Of the 239 diagnostic samples, 51 isolates were ruled as suspected positive (*Tritrichomonas* culture-positive) based on the presence of numerous motile trichomonad protozoan organisms visible microscopically on the wet mount. Isolates with at least five visible flagellate organisms per slide were selected for staining because the preparation of an acceptable fixed slide requires a high concentration of organisms. In total, 29.4% (15/51) of the isolates were successfully stained. Morphologic examination of stained flagellates revealed the presence of organisms with three or four unequally sized anterior flagella, a posterior flagellum, a visible nucleus, and undulating membranes (Figure 3). Table 2 shows that 41.2% (21/51) were from Naledi, 37.3% (19/51) from Kagisano-Molopo, a similar 7.8% (4/51) from Mamusa and Greater Taung, and 5.9% (3/51) from Leekwateemane.

**Table 2:** Distribution of positive cultured *Trichomonas foetus.*

Sample area	Number of analyzed samples	Frequency of occurrence	Occurrence (%)	Cumulative %
Naledi	144	21	41.2	41.2
Mamusa	18	4	7.8	49.0
Kagisano-Molopo	65	19	37.3	86.3
Greater Taung	4	4	7.8	94.1
Lekwateemane	8	3	5.9	100
Total	243	51	100	

### RT-PCR screening

**Figure 4:**
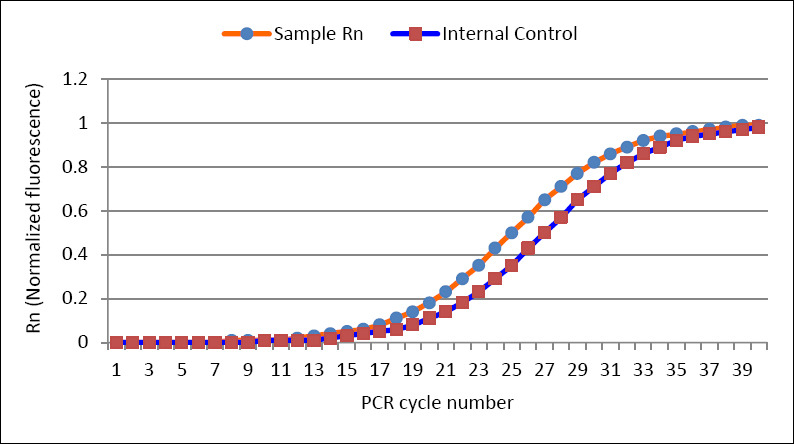
Graphical representation of Rn plotted against the polymerase chain reaction cycle number (Rn is the fluorescence of the reporter dye divided by the fluorescence of a passive reference dye)

RT-PCR identified *T. foetus* in 80.4% (41/51) of the tested samples, indicating a high prevalence of the pathogen (Figure 4). The test revealed an average Ct value of 25.81 (min. 12.38, max. 31.81) with the use of primers specific to amplify *T. foetus* DNA (Figure 4). The RT-PCR primers were based on TFR3/4 primers targeting the ITS region of rRNA. More than 60% of the samples had low Ct values, confirming that the isolate was strongly positive.

### Occurrence and distribution of *T. foetus* based on RT-PCR

**Figure 5:**
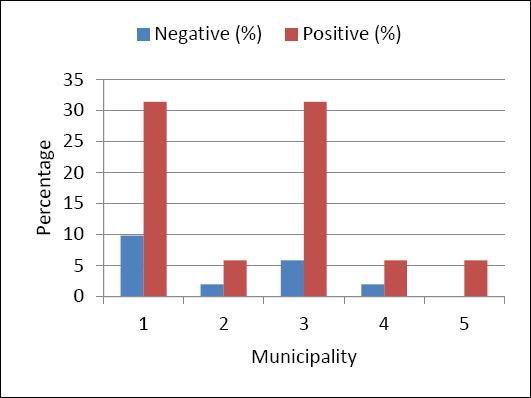
The real-time polymerase chain reaction data distribution of positive amplifications based on 51 tested samples from the different municipalities of Doctor Segomoti Ruth Mompati District in the North-West Province of South Africa.

Figure 5 shows the amplification of *T. foetus* at various levels. Of 51 samples tested by RT-PCR, 80.4% (41) were confirmed as *T. foetus*. The distribution of these positive *T. foetus* isolates was as follows: Naledi – 31.4% (16), Mamusa – 5.9% (3), Greater Taung – 5.9% (3), Lekwateemane – 5.9% (3), and Kagisano-Molopo – 31.4% (16).

### Conventional PCR

**Figure 6:**
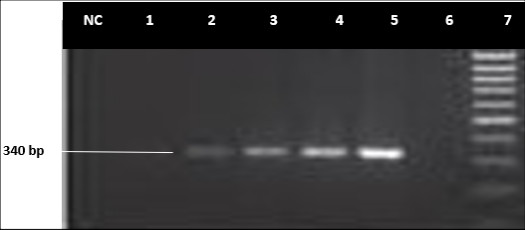
An Agarose gel electrophoresis example of typical polymerase chain reaction amplicons of *Tritrichomonas foetus*. Lane 7-100 bp DNA ladder, Lane 6-negative control, Lane 5-positive control (MT750332.1), Lane 4-positive field isolate (strong band), Lane 2 and 3-positive field isolate (weak bands), Lane 1 and NC = Negative field isolate.

Of the 51 isolates confirmed positive via RT-PCR, 12 (23.5%) were analyzed by conventional PCR and gel electrophoresis with primer pairs TFR1 and TFR2. These results were deemed positive, producing good PCR products suitable for DNA sequencing. Agarose gel electrophoresis showed positive amplification bands for the sample IDs NWT: 6, 12, 14, 18, 20, 21, 23, 25, 28, 30, 37, and 39. The amplicon base-pair regions ranged from 300 to 340 bp, as shown in Figure 6.

### Prevalence of *Tritrichomonas* based on conventional PCR

**Figure 7:**
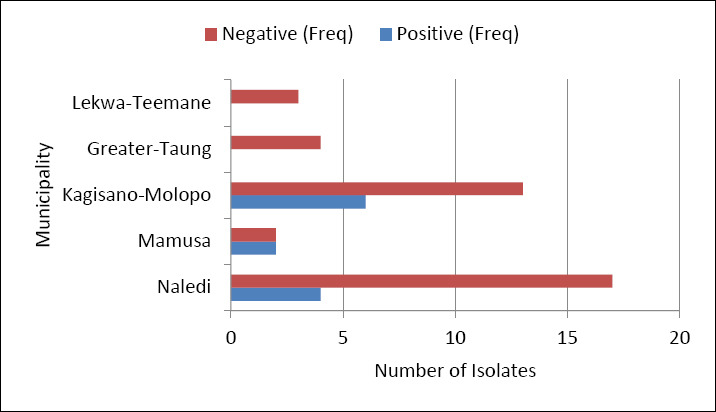
Illustration of the positive amplification data of 51 isolates based on conventional polymerase chain reaction, which revealed positive amplification of samples from three local municipalities: Naledi, Mamusa, and Kagisano-Molopo.

Considering the total number of positive samples (n = 51) and the 12 (23.5%) tested with conventional PCR, the distribution of amplified PCR products was as follows: 7.8% (4/51) from Naledi, 3.9% (2/51) from Mamusa, and 11.8% (6/51) from Kagisano-Molopo local municipalities, as shown in Figure 7.

### Statistical comparison of RT-PCR and conventional PCR

The comparison of the two tests is presented in Table 3. It was observed that 76% (39/51) of the samples were negative by conventional PCR, and 23.1% (9/39) were also negative by RT-PCR. The remaining samples (76.9%, 30/39) tested positive by RT-PCR. Conversely, 23.5% (12/51) of samples tested positive with conventional PCR, and 8.3% (1/12) of these were negative with RT-PCR. The majority (91.7%, 11/12) were positive in RT-PCR.

**Table 3:** Cross-tabulation of *Tritrichomonas foetus* occurrence based on RT-PCR and conventional PCR.

Diagnostic method	Negative, n (%)	Positive, n (%)	Total, n (%)
Conventional PCR	39 (76.5)	12 (23.5)	51 (100.0)
RT-PCR	10 (19.6)	41 (80.4)	51 (100.0)

PCR = Polymerase chain reaction, RT-PCR = Real-time PCR.

Cochran’s Q test (Table 4) was performed to determine the significance level between the two PCR tests. The p-value was less than 0.05, indicating a significant difference in incidence rates between the two PCR techniques. The chi-square test in this study was set at p-value > 0.05 and was conducted to assess the relationship between geographic area and PCR results. The likelihood ratio chi-square p-values were greater than the 5% significance level, indicating no statistically significant association between municipal origin and PCR outcomes for both conventional PCR and RT-PCR (Table 5).

**Table 4:** Summary of statistical values based on Cochran’s Q test.

Test statistics	Value
N	51
Cochran’s Q	27.129
Df	1
p-value	0.000

**Table 5:** Chi-square test results for area versus RT-PCR and conventional PCR.

Chi-square tests	Value	Degree of freedom	p-value
Likelihood ratio Chi-square test: Area versus RT-PCR results	1.857	4	0.762
Likelihood ratio Chi-Square test: Area versus conventional PCR results	5.956	4	0.202

PCR = Polymerase chain reaction, RT-PCR = Real-time PCR.

### Phylogenetic and sequence analysis

The phylogenetic analysis of the *Tritrichomonas* strains used the 5.8S rRNA and ITS 1/2 region genes to determine how closely the strains from this study are related to other isolates from GenBank. We analyzed the data using MEGA X software 5.05 [48]. This study pioneers the phylogenetic characterization of *T. foetus* isolates from South Africa, revealing a distinct Southern African genotype with potential evolutionary links to isolates from Namibia and Australia.

**Figure 8:**
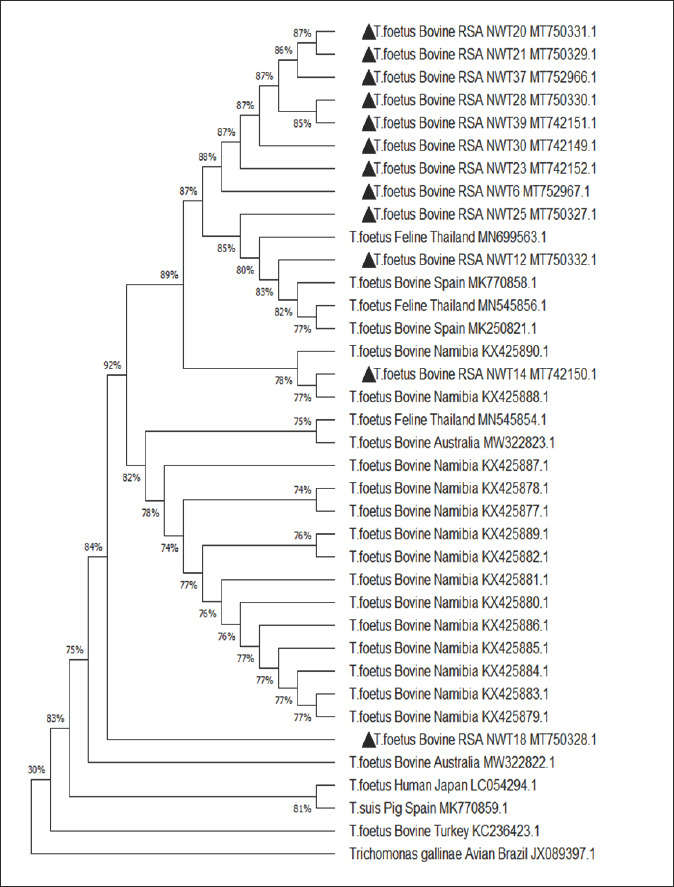
Phylogenetic tree based on the 5.8S rRNA and ITS 1-2 REGION. Genes using the maximum-likelihood and Tamura-Nei model. Bootstrap values greater than 50% from 1000 replicates are shown. The analysis included 37 nucleotide sequences. The Codon positions comprised were 1st+2nd+3rd+Noncoding. There were 375 positions in the final dataset. The out-group was used as *T. gallinae* (JX089397.1). The isolates from the current study are highlighted with a triangle ▲. ITS = Internal Transcribed Spacer.

Figure 8 shows the maximum-likelihood reconstruction (–1321.06). The percentage of trees with associated taxa clustered together is displayed next to the branches. The proportion of sites where at least one unambiguous base was present in at least one sequence for each descendant clade is shown next to each internal node in the tree. The sequenced regions exhibited high sequence similarity (86%–97%) (Table 6).

**Table 6:** Similarity percentages of the identified *Tritrichomonas foetus.*

Sample ID	Reference from NCBI databae	Similarity (%)	Accession number in GenBank	Assigned accession number
NWT-6	*T. foetus*	98	MN545854.1	MT752967
NWT-12	*T. foetus*	100	MN545856.1	MT750332
NWT-14	*T. foetus*	100	MN699563.1	MT742150
NWT-18	*T. foetus*	98	MW322823.1	MT750328
NWT – 20	*T. foetus*	89	KX425890.1	MT750331
NWT – 21	*T. foetus*	89	KX425889.1	MT750329
NWT – 23	*T. foetus*	97	KX425888.1	MT742152
NWT – 25	*T. foetus*	99	KX425887.1	MT750327
NWT – 28	*T. foetus*	87	KX425886.1	MT750330
NWT – 30	*T. foetus*	100	KX425885.1	MT742149
NWT – 37	*T. foetus*	90	MK770858.1	MT752966
NWT-39	*T. foetus*	86	KX425884.1	MT742151

NCBI = National Center for Biotechnology Information.

In cluster 2, one *T. foetus* isolate from this study (MT742150.1) grouped with bovine isolates from Australia (MW322823.1) and the “Southern African genotype” from Namibia (KX425878.1–KX425887 and KX425889), sharing 77%–82% similarity. In cluster 3, another isolate (MT750328.1) grouped with bovine isolates from Australia (MW322822.1) and Turkey (KC236423.1), with 80% similarity. The *T. foetus* isolate from humans in Japan (LC054294.1) and the *T. suis* isolate from pigs in Spain (MK770859.1) clustered together, showing 81% similarity. Differences in nucleotides and cluster positions may reflect mutation, evolution, and adaptation to new environments.

Although zoonotic spread possibilities were suggested, unfamiliar clinical diseases observed in the avian population highlight the ongoing adaptation of new pathogenic isolates of the *Tritrichomonas* species. Additionally, sequence analysis of the 5.8S rRNA and ITS 1/2 regions of all 12 *T. foetus* isolates from this study showed identical sequences (NWT 6, 12, 14, 18, 20, 21, 23, 25, 28, 30, 37, 39) and strong similarity to bovine, feline, porcine, and human isolates of T. foetus reported elsewhere (Figure 8) [28].

## DISCUSSION

### Morphological detection of trichomonads

#### Culture and microscopic examination

In the present investigation, trichomonads were mainly detected through culture and microscopic examination of sheath wash samples from bulls in the DSRM region of South Africa. This study identified organisms with morphological features similar to *T. foetus*. However, it should be noted that the survival of non-*T. foetus* in serial culture passages are rare [49]. It is also reasonable to accept that prolonged transport time and unfavorable temperature conditions during exposure can lead to trichomonad pseudocyst formation [50, 51]. The findings of this study support previous observations as reported by Dufernez *et al*. [52].

#### Modified Giemsa staining and morphologic features

To further investigate the morphological features of the isolates, a modified Giemsa staining method was employed, followed by high-magnification microscopy (×100). This is an unusual procedure for routine testing of bovine trichomonosis in South Africa. The observations in this study showed organisms with one posterior flagellum and three to four anterior flagella of different sizes, stained purple. The undulating membrane also appeared purple-stained, displaying 2–5 waves. A bright purple stain was visible on the axostyle, and the nucleus was stained dark purple (Figure 3). The exact number of flagella was visible on well-stained slides, and some isolates included trichomonads with a fourth anterior flagellum.

### Co-existence of other Trichomonad spp.

Additionally, *T. foetus* coexisted with another suspected *Tritrichomonas* species, *Tetratrichomonas spp*. [28]. According to Dufernez *et al*. [52], *Tetratrichomonas spp*. and *Pentatrichomonas spp*. are commonly found in clinical preputial samples (sheath wash) of bulls, with the number of anterior flagella of the organisms observed microscopically [53]. The findings from the current study further indicate that culture, followed by microscopic detection of motile flagellates consistent with trichomonads, is an inexpensive but not 100% specific method for detecting *T. foetus*. Therefore, more advanced techniques, such as PCR and DNA sequencing, should be used as confirmatory methods for diagnosing *T. foetus* in clinical samples and for identifying unknown flagellates [18, 19].

### Municipality-level occurrence based on morphologic detection

The isolates, based on morphological analysis in the present study, showed an overall detection rate of 21.3% (51/239), with trichomonads unevenly distributed across municipalities in the Dr. Segomotsi Ruth Mompati District. Although microscopic examination of cultured sheath wash samples is not 100% specific for *T. foetus* identification, it reliably detects trichomonads. In this study, many samples tested positive from areas near the international borders of Namibia and Botswana, namely Naledi and Mamusa. This may be due to their proximity to these borders, where previous *T. foetus* detections have been recorded (3.8% in Namibia and 3.33% in Botswana) [36]. Additionally, regular transnational movement of bovines is known to facilitate the spread of diseases [54, 55], which could explain the findings here. The incidence rate was lower in Mamusa (7.8%), Greater Taung (7.8%), and Lekwateemane (5.9%) as shown in Table 2. It is likely that the relatively small sample size of bulls, ongoing testing, and use of AI contribute to the significantly lower occurrence of AI in these areas [27].

### Factors influencing prevalence patterns

#### Local and regional risk factors

The high prevalence of *T. foetus* observed in this study may indicate increased disease transmission, geographical risk factors, or seasonal breeding practices that influence pathogen persistence [56, 57]. However, natural breeding methods and cross-border cattle movements significantly contribute to the risk of bovine trichomonosis transmission in South Africa. Furthermore, breed and ecological differences might explain the variation in *T. foetus* prevalence across South Africa. According to the Department of Agriculture, 40% of the total cattle in South Africa are managed extensively by communal farmers [58].

#### Comparative data from the United States and Europe

According to Rae *et al*. [30], *T. foetus* was detected in 6% of *samples* from Florida; further statistical analysis showed herd prevalence ranging from 1.8% to 27.0%. Variation in management systems across regions reflects different levels of bovine trichomonosis regulation in the United States [21]. This is reflected in Florida’s prevalence estimate of 6.0%, which was notably higher than those reported in studies from Colorado and Nebraska, where <1% (5/2,909) of bulls tested positive using the same protozoal culture method [59].

Bovine trichomonosis is thought to have been eradicated in many EU countries, although it still persists in some areas where AI is not used [60]. In Spain, prevalence based on traditional culture and microscopy was reported as 2.9% [61]. Study of Mendoza-Ibarra *et al*. [20] reported a high prevalence in the Austuriana de la Montaña breed in Northern Spain: 32% in bulls, as determined by microbial culture and PCR. Another retrospective study (2011–2015) in Spain reported an average prevalence of 12.7%. Additionally, Collantes-Fernández *et al*. [23] documented a decline from 17.9% in 2011 to 7.3% in 2015, indicating improved control efforts across 15 Spanish provinces.

#### Data from African countries

In Nigeria, *T. foetus* has been reported in the Bunaji, Sokoto Gudali, Keteku, and Red Bororo breeds [62]. Cows, bulls, and heifers are all susceptible; however, bulls serve as the main reservoir, with older bulls showing greater susceptibility due to more developed epithelial crypts [27]. Younger bulls (<3 years) have undeveloped penile crypts and tend to have lower infection rates [21], but they are not resistant [34]. Bulls aged ≥4 years have been documented as lifelong asymptomatic carriers [26].

Across Africa, the disease is thought to be more common in areas where AI is not widely used [22, 63]. Detection issues come from weak reporting systems and diagnostic problems [57, 64]. In Zimbabwe, bovine trichomonosis was confirmed for the first time using PCR and microscopy, with 5 out of 31 cattle testing positive [65]. Malawi showed no significant prevalence [66], similar to Tanzania (0%) [67]. No cases were found in Swaziland or Zambia, although Botswana (3.33%) and Namibia (3.75%) reported positive cases [36]. In Nigeria, prevalence varies greatly: 14.9% in the South [62], but 0% in northern states [68, 69], likely due to diagnostic constraints and single-sampling efforts [2]. This variability in the prevalence reported has been ascribed to differences in herd management practices, the age structure of bulls, intensity of surveillance, and diagnostic methods, as it has been the case in other areas such as the United States [70, 71].

The actual prevalence of bovine trichomonosis in Africa still remains unknown. Most research depends on microscopy and culture methods, emphasizing the need for more molecular studies.

### PCR findings and diagnostic performance

#### RT-PCR detection rates

The RT-PCR of the suspect-positive samples in this study identified 80% (41/51) as *T. foetus*. Although 10 samples tested negative by RT-PCR, all 51 cultured samples were still considered positive and confirmed using conventional PCR.

#### Value of molecular diagnostics

Overall, the data showed a notably higher occurrence compared to previous South African studies (2.1%–26.4%). This study makes a significant advance by using PCR-based molecular techniques, which provide greater specificity and reliability than traditional methods. According to earlier studies, PCR methods have proven effective at identifying various *Trichomonas* species [72–74].

#### Influence of PCR inhibition

Meticulous inhibitors produced by contaminants can degrade animal and plant DNA [75]. In this study, RT-PCR produced lower-than-expected Ct values (Figure 4). Guerra *et al*. [76] observed lower Ct values with culture enrichment, which is also relevant here. Higher DNA concentrations lead to lower Ct values, indicating stronger amplification.

One sample (NWT18) tested negative on RT-PCR but positive on conventional PCR; sequencing and BLAST confirmed T. foetus. RT-PCR failure may be due to human error, DNA inhibition, or adverse transport conditions [77]. PCR sensitivity in stored samples has been reported to decrease over time [78]. Preputial samples may contain urine, feces, or blood, which can inhibit PCR even at low levels [79, 80]. According to the BLAST results, the DNA sequences of 12 positive amplifications by conventional PCR were deposited in GenBank (accession numbers: MT750327 - MT750332, MT742149 - MT742152, and MT752966 - MT752967). The isolates were 96-100 percent identical to the isolates in The National Center for Biotechnology (NCBI) and GenBank databases, as shown in Table 7.

Conventional PCR analysis (Figure 6) yielded positive amplicons with the primers listed in Table 2. According to Hussien [81], *Trichomonas* species can be differentiated by gel electrophoresis band differences. Data from this study demonstrated a strong correlation between culture and RT-PCR, but Cochran’s Q test indicated a significant difference between the PCR methods (p > 0.05).

### Comparison with previous diagnostic studies

Numerous studies have compared culture, conventional PCR, and RT-PCR [71]. Ondrak [21] highlighted RT-PCR’s superior sensitivity but noted the potential for false positives. Another study reported complete agreement between conventional PCR and culture in 10 T. foetus-positive samples at Onderstepoort [78]. Although RT-PCR is faster and more efficient [27], conventional PCR remains essential for epidemiological and phylogenetic studies.

Findings from this study support the value of confirmatory PCR in verifying T. foetus in cultured samples.

### Phylogenetic insights and global relatedness

#### Southern African genotype

Interestingly, the phylogenetic tree revealed 77%–87% homology to the “Southern African genotype” from Namibia [22]. The current results indicate that these isolates belong to this genotype, although further genotyping is needed. Variations in nucleotide clustering may indicate mutation, evolution, or environmental adaptation [18].

#### Phylogenetic clustering patterns

Another aim of this study was to describe *Trichomonas* from the North-West Province compared to isolates from other regions. The phylogenetic tree (Figure 8) revealed three main groups. Sequence data from this study clustered with *T. foetus* isolated from cats in Thailand (MK770858.1, MK250821.1) and cattle in Spain, with similarity ranging from 77% to 87%.

- Cluster 2: isolate MT742150.1 grouped with Australian bovine isolates (MW322823.1) and the Namibian Southern African genotype (KX425878.1–KX425887, KX425889).
- Cluster 3: isolate MT750328.1 grouped with bovine isolates from Australia (MW322822.1) and Turkey (KC236423.1), showing 80% similarity.
- The human Japanese isolate (LC054294.1) and *T. suis* from pigs in Spain (MK770859.1) clustered together (81% similarity). Opportunistic infection with the cattle/swine genotype has been identified in humans in Japan [82], despite no contact with reservoir hosts, which suggests transmission through contaminated raw foods.

#### Out-group and interpretation

The out-group, *T. gallinae* (JX089397.1), showed 30% similarity to all isolates, confirming that they are distantly related. Clustering indicates potential homology among strains responsible for infections in South Africa. All isolates from this study clustered closely with each other and with multiple global *T. foetus* strains retrieved from GenBank.

## CONCLUSION

This study offers the first comprehensive molecular analysis of trichomonad isolates from bulls in DSRM district of the North-West Province, South Africa. Culture and microscopy detected trichomonads in 21.3% (51/239) of sheath wash samples, with modified Giemsa staining showing typical *T. foetus* morphology, including posterior and anterior flagella, undulating membranes, and axostyle features. RT-PCR showed a high detection rate of 80.4% (41/51), significantly surpassing conventional PCR, which identified 23.5% (12/51) of samples with adequate DNA for sequencing. Phylogenetic analysis indicated that the South African isolates predominantly clustered (77%–87% similarity) with the known Southern African genotype, as well as isolates from Australia, Turkey, and Japan, emphasizing regional circulation and potential global evolutionary connections.

The high prevalence of *T. foetus* in key municipalities bordering Namibia and Botswana highlights how natural breeding systems and cross-border cattle movement influence disease transmission. The results reinforce the need for routine PCR-based surveillance, especially in communal and extensively managed herds where diagnostic limitations lead to under-reporting and silent spread. The discovery of genetically similar strains across Southern Africa also emphasizes the importance of coordinated regional control strategies and cross-border veterinary policies.

This investigation combined several diagnostic approaches, culture, microscopy, RT-PCR, conventional PCR, and DNA sequencing, to offer a highly reliable detection and characterization framework. It is the first study in this region to use a 5′ TaqMan™ RT-PCR assay with MGB, significantly improving diagnostic specificity. The inclusion of phylogenetic analysis provides new genetic insights into circulating trichomonad strains in South Africa.

Some samples showed inconsistencies between RT-PCR and conventional PCR results, likely due to DNA degradation, transport conditions, or PCR inhibitors associated with preputial material. Sampling distribution was uneven across municipalities, and limited sequencing was possible because only 12 isolates produced high-quality amplicons. Additionally, the cross-sectional design prevents assessment of infection dynamics over time.

Future studies should use larger sample sizes across multiple seasons, include both cow and heifer populations, and implement whole-genome sequencing to better identify strain diversity and evolutionary patterns. Long-term monitoring of herds, including replacement bulls, would enhance understanding of transmission cycles. Assessing the impact of cattle movement, communal grazing systems, and potential wildlife reservoirs would also aid regional disease control planning.

Overall, this study confirms that bovine trichomonosis remains a significant reproductive disease threat in the DSRM district and likely beyond. By demonstrating the high prevalence of *T. foetus* and identifying its close genetic relationship to Southern African strains, the findings highlight the urgent need to incorporate molecular testing into national surveillance and herd health programs. Enhancing diagnostic capacity, enforcing biosecurity measures, and promoting AI could significantly reduce reproductive losses in South African cattle systems. This research provides a vital foundation for improved disease management, regional collaboration, and ongoing molecular epidemiological studies of *T. foetus* and related trichomonads.

## DATA AVAILABILITY

The obtained nucleotide sequence data from the current study have been deposited in the NCBI GenBank database and are publicly available as accession numbers, MT750327- MT750332, MT742149-MT742152, and MT752966-MT752967. Additional data used to validate the results of this study, including laboratory work, statistical analysis, and graphical work, are fully included in the manuscript.

## AUTHORS’ CONTRIBUTIONS

MM: Conceived the project. AHS and MET: Sample collection and laboratory work. NL, BGM, and JMDL: Study design, laboratory results interpretation, and drafted and redited the manuscript. All authors have read and approved the final version of the manuscript.
